# Illusory Contours over Pathological Retinal Scotomas

**DOI:** 10.1371/journal.pone.0026154

**Published:** 2011-10-12

**Authors:** Elisa De Stefani, Luisa Pinello, Gianluca Campana, Monica Mazzarolo, Giuseppe Lo Giudice, Clara Casco

**Affiliations:** 1 Department of Neuroscience, University of Parma, Parma, Italy; 2 Paediatric Low Vision Center, Department of Paediatrics, University of Padua, Padua, Italy; 3 Department of General Psychology, University of Padova, Padova, Italy; 4 San Paolo Ophthalmic Center, San Antonio Hospital, Padua, Italy; National Institute of Mental Health, United States of America

## Abstract

Our visual percepts are not fully determined by physical stimulus inputs. Thus, in visual illusions such as the Kanizsa figure, inducers presented at the corners allow one to perceive the bounding contours of the figure in the absence of luminance-defined borders. We examined the discrimination of the curvature of these illusory contours that pass across retinal scotomas caused by macular degeneration. In contrast with previous studies with normal-sighted subjects that showed no perception of these illusory contours in the region of physiological scotomas at the optic nerve head, we demonstrated perfect discrimination of the curvature of the illusory contours over the pathological retinal scotoma. The illusion occurred despite the large scar around the macular lesion, strongly reducing discrimination of whether the inducer openings were acute or obtuse and suggesting that the coarse information in the inducers (low spatial frequency) sufficed. The result that subjective contours can pass through the pathological retinal scotoma suggests that the visual cortex, despite the loss of bottom-up input, can use low-spatial frequency information from the inducers to form a neural representation of new complex geometrical shapes inside the scotoma.

## Introduction

Individuals with loss of foveal vision and consequent loss of bottom-up input to the “foveal” cortex, due to juvenile macular degeneration (JMD), often experience “vision” inside their scotoma; although their central retina has no detectable residual vision, they perceive the surrounding background as expanding, invading and “filling-in” the region of the scotoma [1].

Filling-in has been known for many years in normal-sighted subjects, in physiological scotomas at the optic nerve head [2], and in the artificial scotoma produced by a small region of uniform luminance surrounded by a structured background [3].

It is currently debated whether perceptual filling-in is caused by active neural processes, i.e., activity in visual areas, or whether there is no need for the neural representation of the surface region where the filling-in of visual features is perceived [4]–[7]. Although many electrophysiological studies in animals [8]–[12] and both psychophysical [3], [13]–[15] and fMRI studies in humans [16], [18] suggest that filling-in results from a neural interpolation mechanism, other studies did not find evidence of a neural representation of filling-in [17], [19], which opens the alternative hypothesis that the region of the missing input is ignored and remains unnoticed.

Previous studies on the filling-in phenomenon can be distinguished on the bases of the type of input disturbance (blind spot, artificial scotoma, pathological retinal scotoma and scotoma due to cortical damage). An often-debated issue is whether different mechanisms underlie filling-in at the optic nerve head and at scotomas [20]–[21].

Most studies used simple stimuli (brightness, simple shapes) that produce filling-in of luminance edges or surfaces in the blind spot [13], [22] in the artificial scotoma [23] and in the pathological retinal scotoma [1], [14], [20].

To demonstrate that filling-in is an active visual process involving the creation of an actual neural representation of the surrounding area, complex visual stimuli, such as textures and complex shapes, must be used. Most studies using complex patterns, such as 2-D static and dynamic dot patterns, were carried out in artificial scotoma [3], [24]–[27].

The results of the very few studies that presented complex patterns in the blind spot [28] or in the physically damaged retina [15], [21] suggest that even large central scotomas are perceptually filled in with the surrounding patterns whose perceptual characteristics are compatible with an active process.

**Figure 1:**
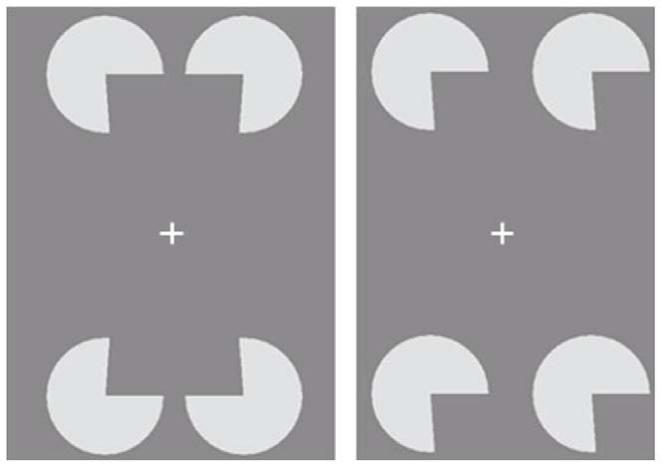
Stimuli. To perceive the illusory gray rectangle in the absence of luminance edges defining its control, the openings of the four white inducers must be specular (left) and form the corners of the illusory rectangle. The figure shows an example of a thin Kanizsa illusory rectangle formed by inducer openings with acute angles. Only the global grouping of the four inducers creates the illusion. As a matter of fact, when the same four inducers are all oriented in the same direction, the illusory shape is not perceived (right).

In the present study, we used as a complex stimulus the well-known Kanizsa [29] rectangle (Figure 1), a figure that is perceived in the absence of physically defined surfaces and luminance edges. The advantage of using this stimulus is that it allows one to ask not simply whether there is interpolation of information across visual space in regions where that information is absent, but also whether perceptual completion phenomena occur inside the scotoma of subjects with juvenile macular degeneration, by which visual patterns not physically presented in the external input are perceived in that region. If illusory figures can be perceived in the central retinal region that has no detectable residual vision, this would possibly indicate that filling-in consists not simply of completion of surrounding features (colour, brightness, motion, texture, and depth) and surface, but of a neural representation of new complex geometrical shapes inside the region that do not receive retinal input.

We assumed that if illusory contours are perceived by an active process, subjects should be able to actively perform complex visual discriminations of the attributes of these contours, such as the disambiguation of slight differences in subjective contour curvature. This result would be relevant for two reasons. First, fMRI showed a retinotopically specific response to these subjective contours (in the absence of retinal stimulation) within the primary cortex [30]–[31], whereas the analogous V1 representation of these contours in the blind spot region in V1 is absent [19]. The demonstration that a precise discrimination of these subjective contours is possible in the damaged retina as in the normal retina but not in the blind spot would suggest that a neural representation of illusory contours occurs in the pathological but not physiological scotoma. Second, it would allow scientists to improve our understanding of the level of central processes involved in filling-in [5]. Indeed, the capability of discriminating slight differences in curvature [19], [30] and spatial position [20] is mediated, at least in part, by low-level cortical processing, and cannot be explained by a representation of the region of visual space falling within the scotoma as being a dark or blurred version of the surrounding area, or having zero contrast.

The Kanizsa figure is perceived every time the corners of the figure are made visible by the presence of “inducer” openings with the appropriate spatial organisation (Figure 1, left). We used four inducers that were white circles of 5° diameter with their either acute or obtuse openings that gave rise to the perception, if disposed specularly, of a thin (with concave sides) or fat (with convex sides) illusory rectangle. As Figure 1 shows, the openings were rotated in such a way that the horizontal illusory contours were straight. It has often been speculated that to perceive the Kanizsa illusory figures, activity from neurons responding to the inducer openings is spread in the direction of the virtual sides of the figure; this allows, in the absence of physical input, edge detectors to be activated along the sides of the illusory figures and surface detectors, with their receptive fields into the illusory surface, to be activated [5].

By presenting the inducers at the borders of the scotoma, it can be proven whether they give rise to the perception of the Kanizsa rectangle with its illusory vertical sides and surface falling inside the scotoma, where there is no detectable residual vision. To assess whether JMD subjects were capable of discriminating the curvature of the illusory Kanizsa figure falling within the scotoma, the discriminability of “real” features presented at the scotoma borders (acute vs. obtuse openings) and the “illusory” features (concave or convex sides) of the Kanizsa rectangle presented inside the scotoma were compared in three JMD subjects and six age-matched controls.

## Results

**Figure 2:**
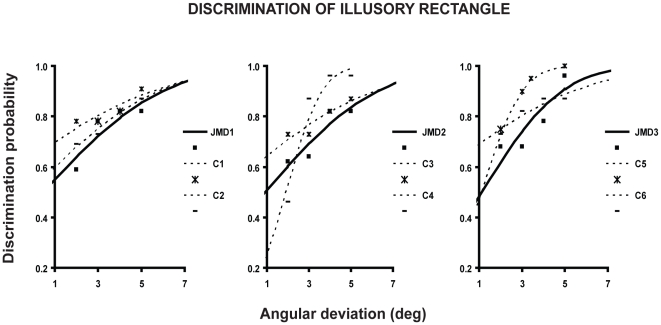
Discrimination of illusory rectangle. Psychometric functions are fit to the probability of discriminating whether the Kanizsa illusory rectangle was thin or fat as a function of the deviation of openings' angles from 90 deg and plotted separately for each JMD subject and his or her two age-matched controls.

In Experiment 1 we measured observers' efficiency in discriminating whether the Kanizsa illusory rectangle was thin or fat. Figure 2 reports the psychometric functions describing the probability of discriminating correctly whether the Kanizsa rectangle was fat or thin as a function of the amount of deviation of the openings sizes from a 90° angle. Clearly, the psychometric functions of the three JMD subjects overlap that of age-matched controls. Chi square with Yates correction [32] revealed that the threshold (see General Method) of each JMD subjects never significantly differed from observed thresholds in the control group (JMD1: x^2^ = 4.4, P = 0.51; JMD2: x^2^ = 4.7, P = 0.45; JMD3: x^2^ = 4.11, P = 0.53).

**Figure 3:**
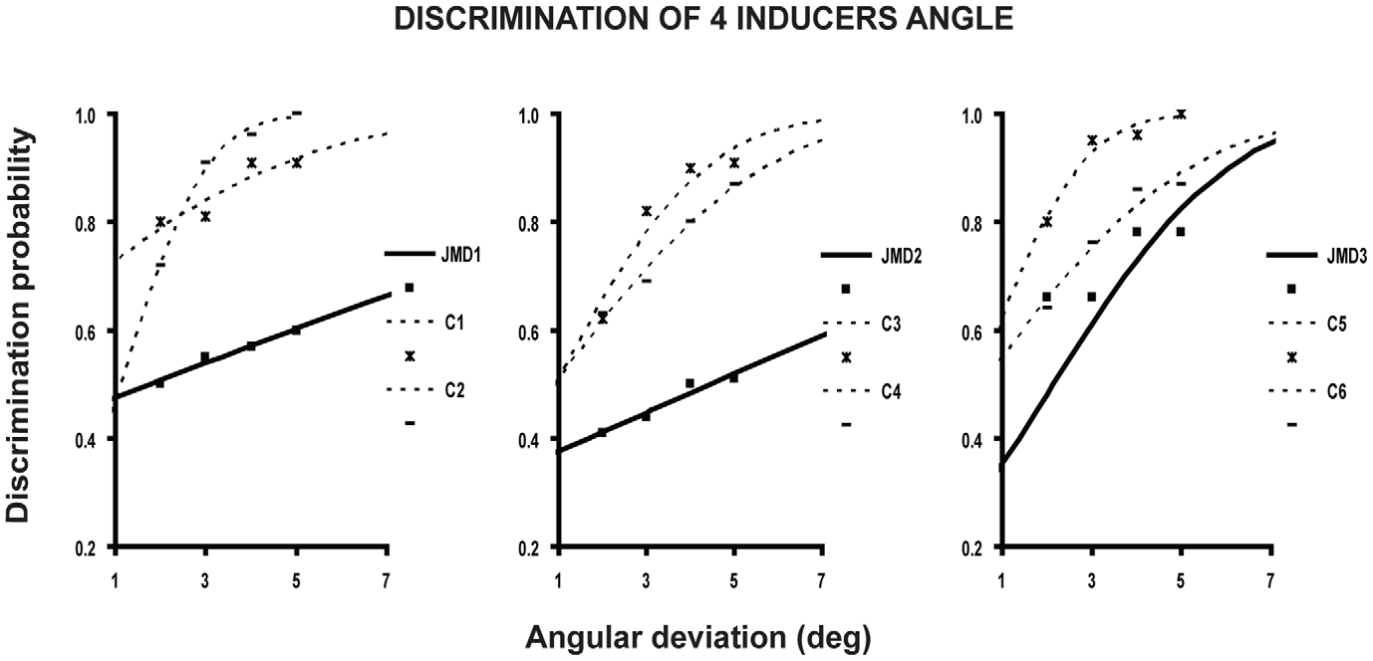
Discrimination of four inducers angle. Psychometric functions are fit to the probability of discriminating whether the openings of the four inducers simultaneously presented were acute or obtuse, as a function of the deviation of openings' angles from 90 deg and plotted separately for each JMD subject and his or her two age-matched controls.

In Experiment 2, we examined how well JMD observers discriminated whether the openings were acute or obtuse. We positioned the inducers with their openings all facing to the right (Figure 1, right). The task of the subjects was to discriminate whether the opening angle was acute or obtuse. The results (Figure 3) were unequivocal: the probability of discriminating the angle increased, in controls, as the angle deviated from 90° and reached ceiling performance at large angles. Instead, except for JMD3, who had some residual vision in the right eye (her visual acuity was 2.5/10), JMD1 and JMD2 were incapable of performing the task and behaved at chance regardless of angle size. Chi square with Yates correction revealed that the threshold of two JMD subjects significantly differed from observed thresholds in the control group (JMD1, x^2^ = 12.4, P = 0.03 and JMD2, x^2^ = 13.7, P = 0.02). JMD3′s threshold did not differ from those of the controls (x^2^ = 7.02, p = 0.22).

To test whether simultaneous presentation may facilitate the task in controls, because of probability summation, only one inducer was presented randomly in one of the four random positions.

**Figure 4:**
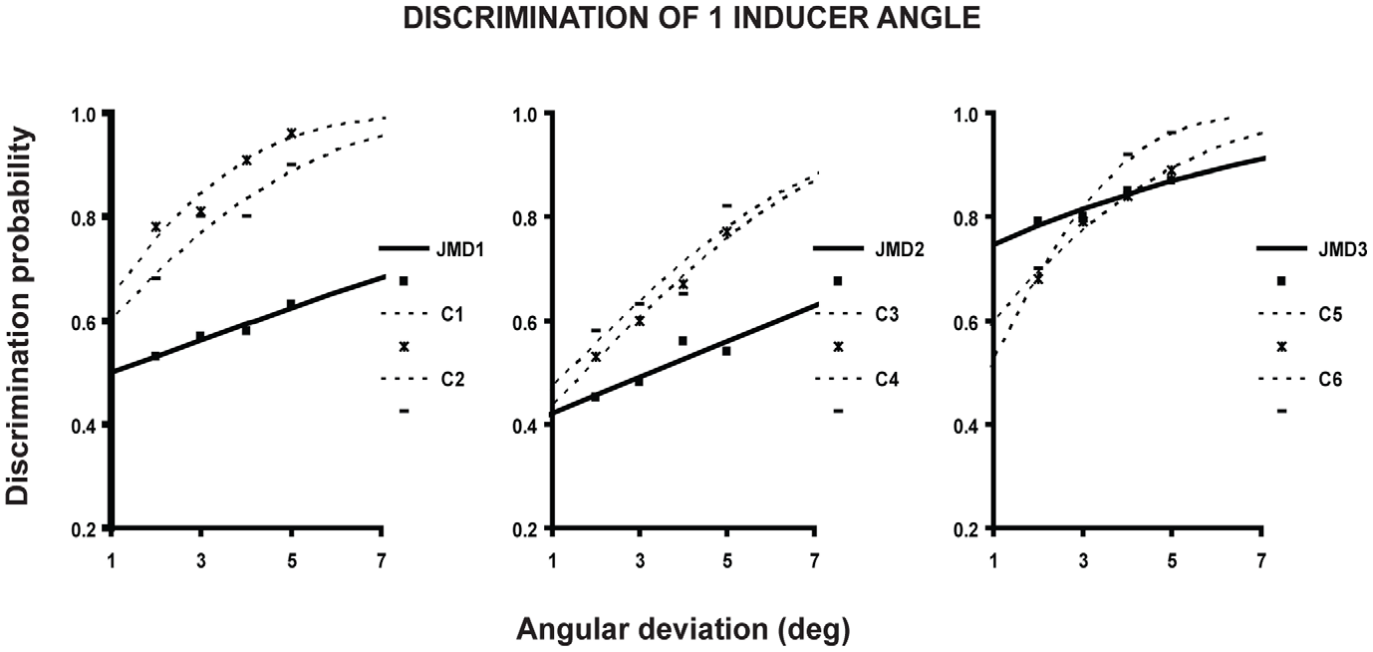
Discrimination of one inducer angle. Psychometric functions are fit to the probability of discriminating whether the opening of one of the four inducers randomly presented was acute or obtuse as a function of the deviation of openings' angles from 90 deg and plotted separately for each JMD subject and his or her two age-matched controls.

Results (Figure 4) replicate those obtained when the four inducers were presented simultaneously. The ANOVA results (with Greenhouse–Geisser correction) revealed that, in the control group, thresholds did not differ in the three experiments [*F* (2, 10)  = 1.79, *P* = 0.25]. Chi square with Yates correction instead revealed that the threshold of two JMD subjects significantly differed from the observed thresholds in the control group (JMD1, x^2^ = 12.4, P = 0.03 and JMD2, x^2^ = 13.6, P = 0.02). However, JMD3′s threshold did not differ from those of the controls (x^2^ = 4.65, P = 0.46).

## Discussion

Overall, the results of the three experiments show that JMD subjects could discriminate slight differences in curvature of illusory contours falling inside the scotoma. This occurs despite the fact that they have reduced resolution with respect to controls for the openings inducing the illusion that were presented in the retinal region surrounding the scotoma, both when only one or four inducers at a time were presented. This last result excludes that JMD subjects judged the orientation of local features, i.e. angle size of the openings, instead of judging whether the sides of the Kanizsa rectangle were fat or thin.

The finding that JMD patients have low resolution for the inducers presented in the surrounding of the scotoma was expected because it is well known that people with macular disease often demonstrate poorer performance on tasks involving discrimination of stimuli presented behind the boundary of the scotoma [33]. Nevertheless, these inducers contribute to the formation of a neural representation of the Kanizsa figure. This provides strong evidence of an intact neural process of filling-in, in the retinal region of absent vision. This process may be triggered by the inducers whose integration across cortical distances could be mediated by long-range horizontal connections [34]. A similar mechanism of active filling-in may lead patients to report [35]–[36] the distance between illusory contours or the distance between inducing circles shorter than it is and also to ignore their own scotoma. Most importantly, it allows to perceive a whole shape (instead of four isolated inducers) and to judge accurately the distortion of its contour. This cannot be done without assuming the coding of the inducers' configuration as a whole shape.

It is worth considering alternative accounts of our results. One is suggested by the finding that the reported position of short line segments is strongly biased toward the interior of an artificial scotoma [34]. However, such a bias would lead to perceive more easily the inwards curvature of the illusory contours and this would favour “thin” but not “fat” judgments. In sum, the most likely interpretation of our results is that the coarse (low spatial frequency) information from the inducers (that does not allow discriminating the angle size of their openings) can instead be used to trigger a filling-in process and form a neural representation of the illusory figure.

The present results are relevant because the majority of macular damage literature is done in seniors with AMD. Filling-in may be affected by the aging process.

It is interesting to speculate whether active filling-in inside the scotoma is indicative of cortical plasticity. The use of low-spatial frequency channels may or may not be connected to plasticity. It has been shown that the inducers centrally presented produce a stronger response in the primary visual cortex when giving rise to illusory contours than when they did not [30]–[31]. On the other hand, Maertens and Pollmann [19] could not find evidence that illusory contours pass through the “blind spot”. They attribute this specific performance deficit to the failure to build a representation of the illusory rectangle in the absence of a cortical representation of the “blind spot”. Since we found that illusory contours are instead perceived when the scotoma results from retinal damage, this suggests that a neural representation of contours can be formed in the retinotopically specific visual cortex, although—because of retinal damage—neurons do not receive retinal input. This is possible when we assume that these neurons, deprived of visual input, are activated by the input outside the scotoma. Indeed, it has been shown that the position, size, and shape of the receptive field of some cortical neurons can change dynamically, in response to artificial scotoma conditioning [23], [34], to retinal lesions in adult animals [37]–[43], and to vision loss in humans [44]–[48]. In the physically damaged retina, the mechanism underlying filling-in may be one aspect of this general cortical reorganisation process that causes a receptive field expansion, in anesthetized cat, when the surround of the receptive field is stimulated but the receptive field itself is not [43]. This plasticity phenomenon may result from disinhibition and consequent unmasking of long-range, normally inactive facilitatory influences between cells with their receptive fields inside the scotoma and those at the boundaries of the scotoma. However, the question of whether the cortical reorganisation hypothesis may explain the filling-in phenomenon is difficult to approach. Indeed, cortical reorganisation is not always found [42], [49] and, when found, it has been shown to depend on task [46], on the position of retinal stimulation [50], and on whether retinal loss is complete [45]. Nevertheless, it provides strong psychophysical evidence that filling-in results from an active process.

The results of the present study may assist in clarifying the underlying neural mechanism by which JMD subjects acquire enhanced responses, not only inside the scotoma but also to complex, attention-demanding visual stimuli in the periphery [45], [51]–[52] and, in particular, in one region that becomes a “new fixation” centre (the “Preferred Retinal Locus”, or PRL) [50]. Understanding filling-in mechanisms may also have important implications for the visual rehabilitation of visually impaired individuals. It may help in the development of visual training programs and creation of visual enhancement techniques that use filling-in processes to improve the visibility of input patterns for people with macular disease.

## Materials and Methods

### Subjects

The experimental sample was composed of three participants (two males and one female) aged 12 to 18 with JMD at the beginning of the study. Patients with bilateral central retinal lesions had a diagnosis, confirmed by an ophthalmologist, of Stargardt's disease (JMD1) or chorioretinal macular scar due to congenital toxoplasmosis (JMD2 and JMD3), involving the central vision. JMD individuals had at least 3-year histories of vision loss and underwent complete ophthalmological examinations.

Best-corrected visual acuity (BCVA) for both eyes was measured with the modified Early Treatment Diabetic Retinopathy Study charts (ETDRS). JMD subjects' BCVA was reduced both in the right and left eyes to 1/30 and 1/10 (JMD1), to 1/10 in both eyes (JMD2), and to 2.5/10 in the right and 1/10 in the left eye (JMD3) (Table 1).

**Table 1:** Patients' characteristics.

Participant	Gender	Age (years)	Visual acuity		Diagnosis	Scotoma size (deg)		Stimulus size(deg; w*h)
			OD	OS		OD	OS	
JMD1	M	18	1/30	1/10	Stargardt'sdisease	11×10	11×10	11×7
JMD2	M	12	1/10	1/10	Congenital toxoplasmosis	9.5×10	14.5×10	11×7
JMD3	F	14	2.5/10	1/10	Congenital toxoplasmosis	5.5×6	9.5×10	11×7

Each JMD individual was matched with two control participants of the same age. In total, the control group was composed of three pairs of healthy subjects (four males and two females) of ages matching each JMD subject, all with normal or corrected-to-normal visual acuity. They had no evidence of ophthalmological diseases on fundoscopy. All subjects were unaware of the purpose of the experiment.

### Ethics Statement

The experiments were approved both by the ethical committee of the Department of General Psychology at the University of Padua and by the Institutional Ethics Committee of the Hospital of Padua. The study followed the tenets of the Declaration of Helsinki. Before testing, all participants gave their written consent.

### Retinal microperimetry and visual field plotting

JMD participants were carefully tested to determine the location of the subject's PRL, fixation stability and visual field loss. A NIDEK MP1 retinal microperimeter was used to map the location of the PRL, and to measure the stability of fixation at the PRL for JMD participants.

To document visual field loss, measurements were conducted using a Goldmann perimeter, a simple test to estimate kinetic perimetry.

Each eye was tested separately. Participants were instructed to maintain fixation with their PRL on a fixation point at the centre of the screen, while a 1° target was moved across the screen. In the first exploration phase, subjects were asked to report whenever the target disappeared. When the scotomatous areas were located, the target was placed inside the scotoma and moved from nonvisible to visible regions (kinetic perimetry). The point of first seeing the target, as reported by the participants, was marked as the edge of the scotoma. Once the scotoma was mapped, targets were presented in random positions in the centre of the scotoma in a search for any residual central vision.

**Figure 5:**
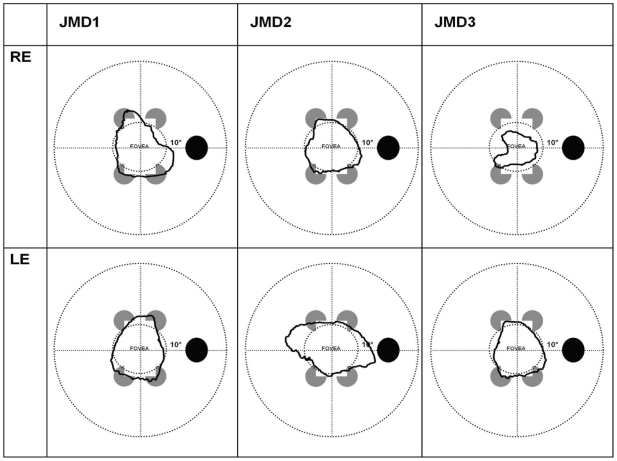
JMD subjects macular lesions. Schematic of visual fields in the right and left eyes of each JMD participant, showing the large extent of the blind field (macular lesion). Black line delimits the damaged area (scotoma).

JMD1 had a dense central scotoma, including all the macula in the right eye and, in the left eye, a 11×10° dense scotoma displaced temporally within the macular area. JMD2 had a 9.5×10° scotoma in the right eye and an elongated 14.5×10° scotoma in the left eye. JMD3 had a smaller scotoma in the right eye (5.5×6°) compared to the left eye (9.5×10°) (Figure 5).

**Figure 6:**
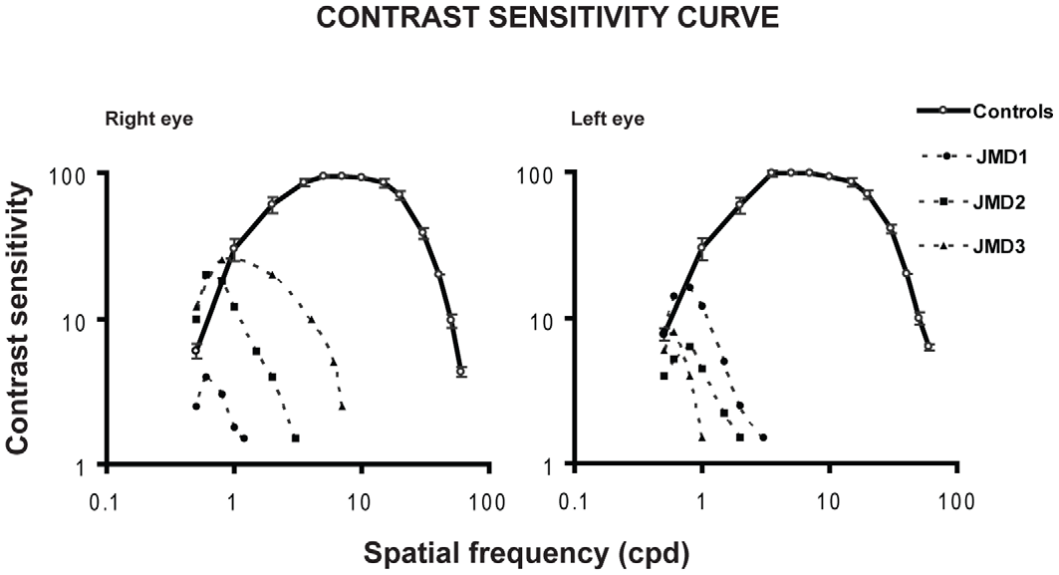
Contrast sensitivity curve. Contrast sensitivity curves of the right and left eyes are measured for spatial frequencies ranging between 0.5 to 60 cycles × degree (cpd). The curves of each JMD participant (filled symbols) are compared with the average contrast sensitivity of the six controls (empty symbols). Error bars indicate SD for the control data.

For each subject (JMD subjects and controls), contrast sensitivity (CS) for spatial frequencies (SF) ranging from 0.5 to 60 cycles × degree (cpd) was measured using Pelli-Robson Charts [53]. Figure 6 compares the CS of the three JMD subjects with the average CS of controls, separately for the left and right eyes. CS functions of JMD subjects showed complete vision loss for SF larger than 3 cpd. The same occurred in the right eye except for JMD3, who could detect high contrast gratings of 6 cpd.

### Apparatus

Stimuli were presented to subjects binocularly on an ACER M715 computer located at a 57-cm viewing distance. Stimuli were generated by the E-prime program and projected centrally on a 33.3×20.7 cm monitor (resolution of 1280×1024 pixels). The refresh rate was set at 60 Hz, and the luminance of the background was 13.34 cd/m2. The study was composed of three separate experiments, the details of which are described in turn.

### Stimuli

Stimuli were composed of four inducers (white-filled circles with an opening, see Figure 1). Each inducer had a diameter of 5°. The distances between the centre of the inducer and the central axes of the monitor were 3° (from the vertical axis) and 6° from the horizontal axis. Inducer luminance was 92.48 cd/m^2^, and background luminance was 13.34 cd/m^2^.

Inducers varied in the angular size of their opening, which were either larger or smaller than 90°: angles smaller than 90° (85, 86, 87 and 88°) created a thin rectangle, whereas those larger than 90° (92, 93, 94 and 95°) gave rise to the perception of fat rectangle. The four inducers were positioned specularly in the main experiment and with their openings all facing to the right in Experiment 2 (Figure 1). In Experiment 3 only one of the four inducers, randomly chosen, was presented.

### Tasks

Subjects were involved in a binary choice task, in which they had to indicate whether the sides of the illusory rectangle were thin or fat, in the main experiment, and whether the inducer openings' angle was acute or obtuse, in experiments 2 and 3.

### Fixation

To ensure that subjects fixated centrally, they had to indicate during the inter-trial interval whether they perceived the gray central fixation cross (line width 1 arcmin), which could not be detected in the central vision by any of the JMD subjects. The trials in which JMD subjects said “yes” were excluded.

### Procedure

Each trial began with the fixation cross presented centrally within two lateral white lines (7.5° distant) for 1500 ms, followed with no interval by the stimulus that was flashed centrally for 100 ms. This short exposure was chosen to avoid eye movements. After the presentation of each stimulus, subjects responded by pressing the appropriate key that triggered the next trial. The entire experiment consisted of 80 randomly presented trials (10 trials for each level of the opening's angle size). Subjects were encouraged to take breaks between trials to maintain high concentration and to prevent fatigue. Subjects could familiarise themselves with the task and stimuli by means of a short training session (8 trials), where stimuli were displayed for 3000 ms. Auditory feedback was given only in practice trials.

### Psychophysical method and data analysis

Psychometric functions were fit, by Probit analysis [54], to the probability of a correct discrimination as a function of the opening's angle. From the psychometric function, we derived thresholds, defined as the smaller deviation from the 90° angle that produce .75 probability of correctly discriminating either between the fat and thin illusory rectangle or between the acute and obtuse opening angles. In each experiment, Chi squared with Yates correction was used to compare thresholds obtained by each JMD subject with thresholds obtained by the six controls. Data from control subjects in the three experiments were analysed using repeated measures ANOVA. The sphericity of the data was evaluated with Mauchly's test [55]. When the sphericity assumption was not supported by that test, Greenhouse–Geisser correction was applied. Since the data for the two orientations were not statistically different, they were not treated separately. Post-hoc pair-wise comparisons were computed with Bonferroni's correction. Alpha level was set at .05 for all statistical tests.
